# Improving Photosynthetic Metabolism for Crop Yields: What Is Going to Work?

**DOI:** 10.3389/fpls.2021.743862

**Published:** 2021-09-21

**Authors:** Matthew J. Paul

**Affiliations:** Plant Science, Rothamsted Research, Harpenden, United Kingdom

**Keywords:** photosynthesis, crop yield improvement, source-sink, Rubisco, cereals

## Introduction

Photosynthesis is an indispensable process that provides the oxygen we breathe, regulates climate, and drives biological processes including crop yields. An important and obvious challenge for crop improvement is: Is photosynthesis and its regulation in crops amenable to improvement to stimulate yields? If so, how could this be done? Photosynthesis is a well-studied process with models of the limitations in the photosynthetic pathway (Zhu et al., 2010). The past 10 years have seen a major effort into improving photosynthesis with a justification that improvements in crop yields need to be quick and large because of the yield plateauing of major crops. The harvest index has been optimized, and therefore, the next bottleneck is proposed as photosynthesis. However, crop yields are yet to benefit from this research despite some tantalizing examples of genetic interventions in models (Arabidopsis and tobacco) including field studies (Glowacka et al., 2018; South et al., 2019). The focus on photosynthesis has been controversial given long-standing evidence with well-reasoned arguments going back to the 1970s that carbon input is not limiting for crop growth and yield (Sinclair et al., 2019). Failure to produce tangible benefits in crops so far from the photosynthetic research effort is attracting gathering criticism (Araus et al., 2021) with arguments for a more balanced approach (Reynolds et al., 2021). However, a recent study overexpresses Rubisco in paddy rice which increases yield in the field under good nitrogen supply by 17–28% (Yoon et al., 2020). This appears to be the first successful direct targeting of photosynthesis in the field in a major food security cereal crop. This article gathers information from recent literature on the photosynthetic improvement of both heavily reductionist approaches and broader-based strategies to provide a balanced opinion of the way forward for the photosynthesis field for crop yield improvement.

## Photosynthesis as Part of a Regulated System

Biological systems consist of parts that make up a whole system. These parts such as photosynthesis need to be considered in themselves to understand the reductionist fundamental molecular science of their makeup and function. This has given rise to photosynthetic models of limiting steps in the process where improvements could be made (Zhu et al., 2010). However, of all biological processes within the plant, photosynthesis is perhaps the most intimately integrated into the system. Therefore, it could be argued that photosynthesis in terms of increasing crop yields cannot be seen like the engine of a motor car where improvements in one or several components could make the engine drive faster or consume less petrol. This is because “the car” in biological terms is not just driven by the engine, photosynthesis, but the car (rest of the plant) strongly interacts and regulates the engine itself. Crop yield is a product of the whole system, not just photosynthesis. So, photosynthesis provides the carbon and energy on which the entire system depends, but this interaction is not linear and depends on numerous factors such as development, leaf and canopy structure and architecture, source-sink feedback, and how photosynthesis is affected within a crop stand in the field. Further, photosynthesis and growth are strongly regulated by the field environment that is significantly different from the laboratory or greenhouse. Photosynthesis is strongly affected by such interactions and feedbacks, such that knowledge of individual components as limiting factors for yield becomes almost lost and meaningless among the noise of the system. Sinclair et al. (2019) made a particularly strong case that long-standing evidence shows that past crop yield increases are not associated with increased photosynthesis. For numerous crops, there has been no rise in carbon exchange rate per unit leaf area. The conclusion was that yield was a multifaceted outcome of many resources and processes; photosynthetic input was almost never the critical variable limiting yield (Evans, 1994; Boote and Sinclair, 2006).

## Transgenic Paddy Rice Overexpressing Rubisco Increases Yield in the Field

A recent article (Yoon et al., 2020) goes against this prevailing view that increasing the photosynthetic rate will do nothing for crop yield. Rubisco was overexpressed in rice with its own promoter. Convincing increases in filled spikelets gave grain yields up to 28% higher in paddy fields. The total biomass was also higher (11–23%). Effects were greatest at nitrogen application rates 100–170 kg per hectare peaking at 28% higher yield at 141 kg nitrogen per hectare. This resulted in more yield gain for nitrogen added, indicating that nitrogen use efficiency was increased. The variety Notohikari has been used since 1985; so potentially, an older variety like this could be more amenable to improvement. However, 6 tonnes per hectare in wild type is a good rice crop yield.

## The Crucial Consideration of Nitrogen and Water for Photosynthetic Improvement

There may be two factors in this study to explain the success of this genetic intervention, specifically the role of nitrogen and water. The main conclusion from Sinclair et al. (2019) was that yield increases are closely dependent on nitrogen accumulation as an essential and quantitative component of seeds. Carbon accumulation in the absence of additional nitrogen does not increase yield. Overexpression of Rubisco in Yoon et al. (2020) increased nitrogen uptake and content particularly just before full heading and through the ripening stages. This could fulfill the requirement laid down by Sinclair et al. (2019) of nitrogen to accompany carbon. As Rubisco is such a large component of leaf nitrogen, increasing photosynthesis by targeting other photosynthetic proteins might not increase nitrogen uptake. Second, increases in photosynthetic gas exchange are normally attenuated by water availability, as much of agricultural production is rainfed rather than irrigated. Intermittent drought can limit yields to at least to some extent each year. The challenge faced is that enhanced photosynthesis leads to more rapid depletion of water. Cross-scale system modeling through the crop life cycle by Wu et al. (2019) shows that enhanced photosynthesis improves biomass gain early in the season when soil water is more abundant, but depletes soil water, leaving less for later crop development. This impinges yields in all but the most hydrated agricultural environments. Yoon et al. (2020) performed their work in a rice paddy where water is not limiting, and hence, photosynthesis is not likely to have been compromised. The filling of existing spikelets was improved, which depends on high photosynthesis later during the life cycle. Therefore, at a system level, both the major limiting factors to crop yield, nitrogen and water, were able to match the enhanced carbon uptake.

## Bespoke Photosynthetic Improvement?

It is possible that overexpressing Rubisco may only work where water and nitrogen availability and uptake will not hold back the benefits of extra carbon, such as in paddy rice. Overexpressing Rubisco where water supply limits yield even to a small extent may provide little or no worthwhile benefit to yield according to Wu et al. (2019). Hence, paddy rice or heavily irrigated crops only may benefit. Intervention in photosynthesis may need to be tailored to environmental conditions. Protection measures such as against reactive oxygen species may be beneficial in situations where water limits yield, as shown in maize in the field (Simmons et al., 2021). Recovery from photoprotection may also be amenable to transgenic intervention, as shown in tobacco (Kromdijk et al., 2016). This approach will not increase the yield potential but enables the existing photosynthetic potential to cope better with insufficient water. Photoprotection may be particularly beneficial in very sunny environments. In a recent study involving the wheat high biomass association panel (HiBAP) conducted in Mexico, Joynson et al. (2021) showed marker-trait associations relating to leaf pigment content that could prevent the propagation of free radicals to improve radiation use efficiency and yield. Selection or genetic intervention in photoprotection may not involve large interactions with the whole system and, therefore, may be relatively straightforward compared with other strategies. However, other approaches that do alter the whole system like optimizing source-sink interactions could potentially benefit crops for both yield potential and abiotic stress resilience.

## The Crucial Consideration of Source-Sink Interaction to Improve Photosynthesis

There are examples where the promotion of sink strength increases photosynthesis and carbon gain for yield. Maize expressing a rice trehalose phosphate phosphatase gene in phloem tissue in developing cobs had higher yield at a range of water availabilities in extensive field trials, at 9–49% higher than wild type with no or mild drought and 31–123% higher with more severe drought (Nuccio et al., 2015). Lower trehalose 6-phosphate (T6P) in phloem tissue enhanced SWEET expression resulting in stronger movement of sucrose into kernels improving kernel set (Oszvald et al., 2018). This genetic intervention prevented the normal decline of photosynthesis in associated leaves over time giving almost 50% greater photosynthetic rates at specific time points showing that stimulation of sink can exert a strong pull on the source. Beechey-Gradwell et al. (2020) overexpressed diacylglycerol acyltransferase to drive triacylglycerol biosynthesis in *Lolium perenne*. High lipid *L. perenne* plants not only accumulated lipid but also had up to 28% higher photosynthetic rate. Diversion of carbohydrates into a lipid carbon sink may sequester carbon away from carbon-sensing mechanisms. This could mitigate the signals that would normally downregulate photosynthesis as part of carbon and energy metabolic homeostasis, meaning that photosynthesis is “blind” to carbon accumulation and can carry on unimpeded while carbon accumulates. This is another example of the strong effect sink can have on source as a consideration in improving photosynthesis. Source-sink modifications for cereals could work where photosynthesis is maximized around grain set before anthesis to establish a large sink through floret initiation and retention. This large sink may then sustain photosynthesis during grain filling (Reynolds et al., 2021). Optimizing canopy structures such as upright leaves to allow more light interception is an approach shown to work to increase wheat photosynthesis to maximize grain set (Richards et al., 2019). Lines with erectophile leaves had a 13% higher yield. Griffiths et al. (2020) showed that effects of drought pre- or post-anthesis affect grain yield parameters differently. Yield is strongly related to grain number when drought is pre-anthesis but has a stronger element of grain size as a yield determinant when drought is applied at anthesis. Environments with intermittent drought need to ideally include germplasm with elements of tolerance to both pre- and post-anthesis drought that also have good yield potential. There were only three such lines out of 150 that fulfilled this requirement (Griffiths et al., 2020). It is proposed that genes that enable high yields in all three conditions may be genes that coordinate source and sink. Source-sink coordination at crucial times in development may be important. Wang et al. (2019) and Wang et al. (2020) showed that genetic regions could be identified containing genes for high source and high sink that could be combined in breeding. For different crops where water is often limiting unpredictably, combining beneficial source and sink genes is likely to be a way forward.

## Conclusion: Ways Ahead for Photosynthesis

Transgenic crops with modified photosynthesis that successfully increase yield in the field in major food security cereals may be rare. The example of Yoon et al. (2020) may have worked because of the special paddy conditions in that experiment. In more water-restricted environments, overexpressing Rubisco may be less successful, and consideration of photoprotection could offer some benefits. Strategies to optimize source-sink would represent a promising way forward given the significant effects that a strong sink can exert on photosynthesis and the strong determination of grain number and also grain size on the source and yield. A stronger sink strength can increase yield with and without drought (Oszvald et al., 2018). Creating a stronger sink strength has the advantage over photoprotection that yield potential is increased too. It may be possible to identify genetic regions for the strong source and sink through GWAS (Wang et al., 2019, 2020). More than anything, improving crop photosynthesis in the field will require larger-scale collaborations involving expertise from experts in institutes and universities, CGIAR centers, and the private sector. This will require adopting strategies critically assessed as likely to work in field conditions by all stakeholders.

## Author Contributions

The author confirms being the sole contributor of this work and has approved it for publication.

## Funding

Rothamsted Research receives strategic funding from the Biotechnological and Biological Sciences Research Council of the United Kingdom.

## Conflict of Interest

The author declares that the research was conducted in the absence of any commercial or financial relationships that could be construed as a potential conflict of interest.

## Publisher's Note

All claims expressed in this article are solely those of the authors and do not necessarily represent those of their affiliated organizations, or those of the publisher, the editors and the reviewers. Any product that may be evaluated in this article, or claim that may be made by its manufacturer, is not guaranteed or endorsed by the publisher.
